# Supplementary figures and images for: Depletion of SMN protein in mesenchymal progenitors impairs the development of bone and neuromuscular junction in spinal muscular atrophy
Source: eLife. 2024 Feb 6;12:RP92731. doi: 10.7554/eLife.92731 (PMC10945524; doi:10.7554/eLife.92731)

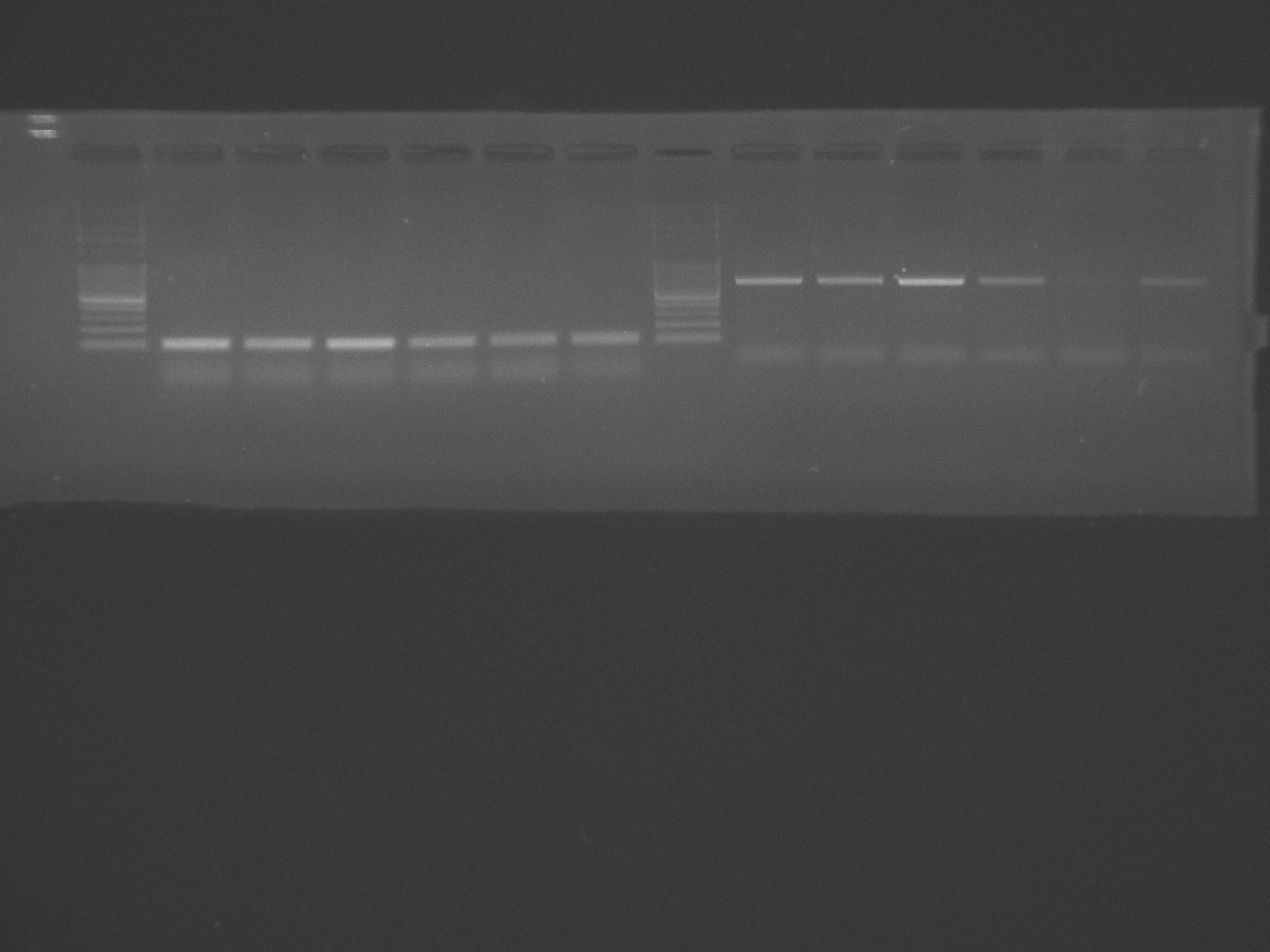

Supplement: Figure 2—source data 1. [file elife-92731-fig2-data1.zip › Figure 2G_Cre.tif]

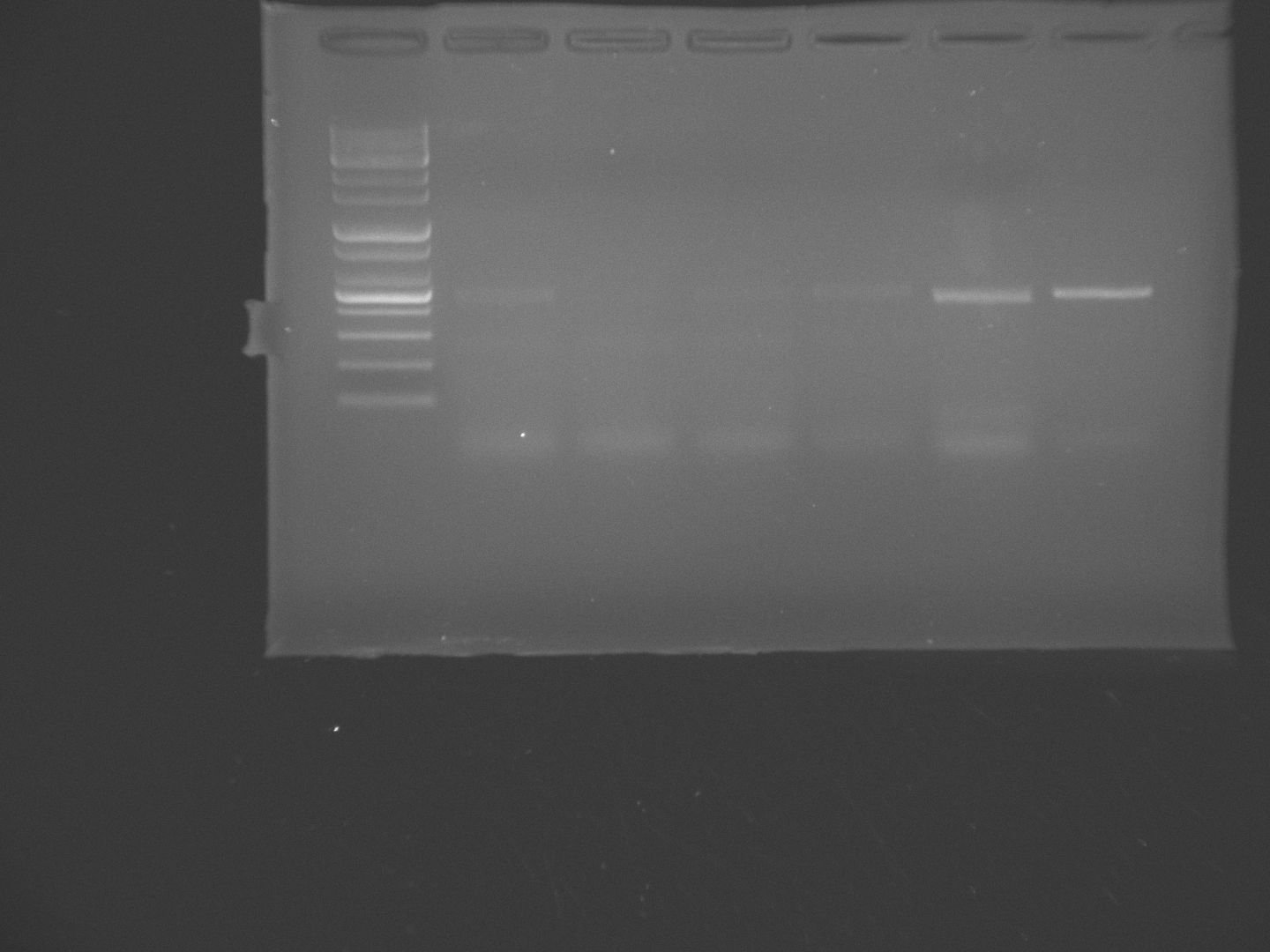

Supplement: Figure 2—source data 1. [file elife-92731-fig2-data1.zip › Figure 2G_Smn1 D7.tif]

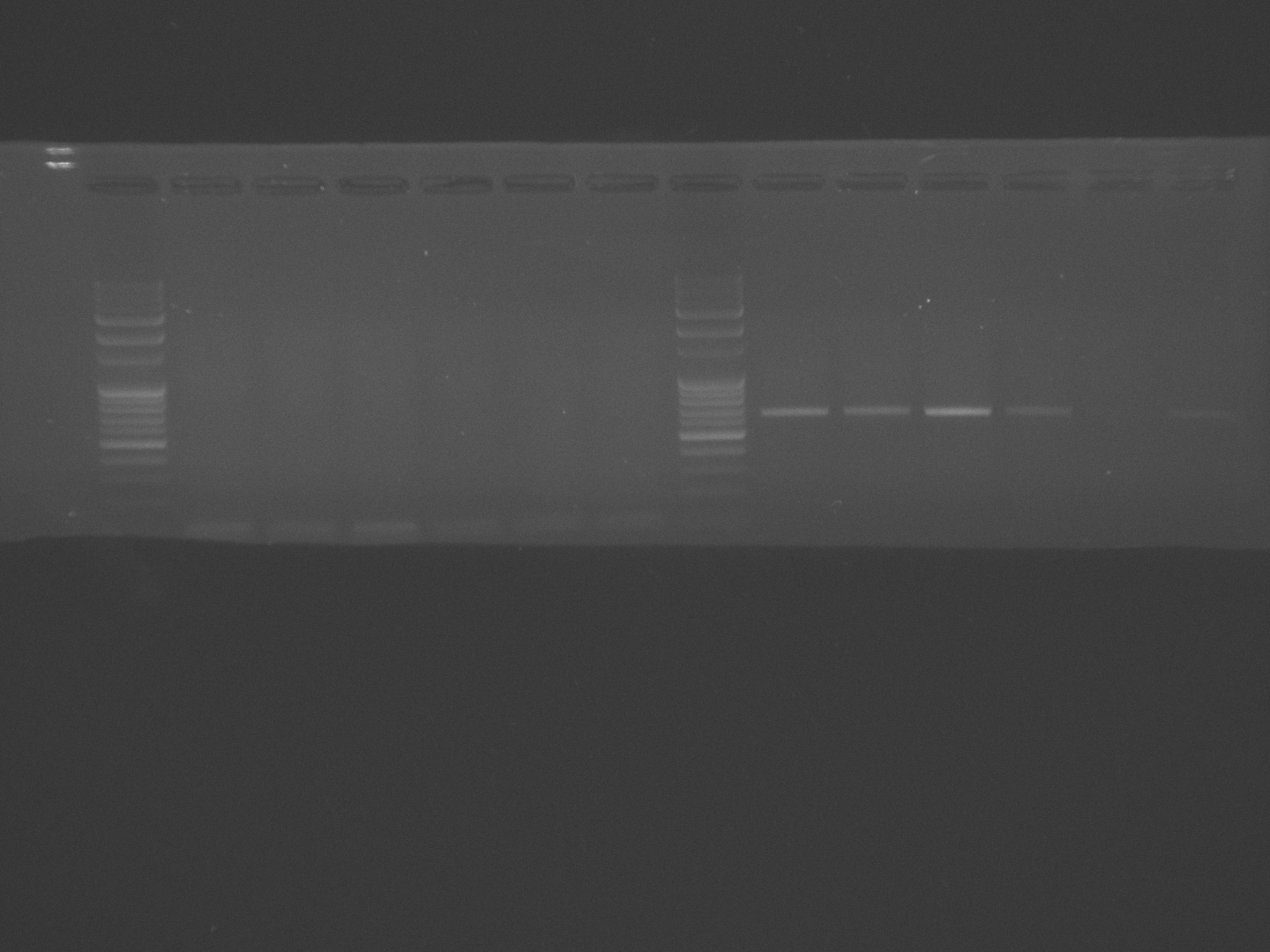

Supplement: Figure 2—source data 1. [file elife-92731-fig2-data1.zip › Figure 2G_Smn1 F7.tif]

**G**

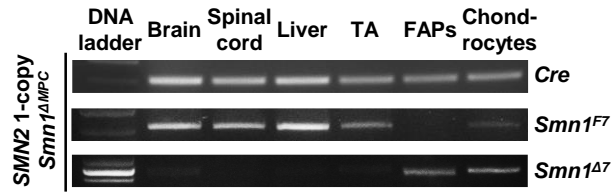

Figure 2G\_Cre

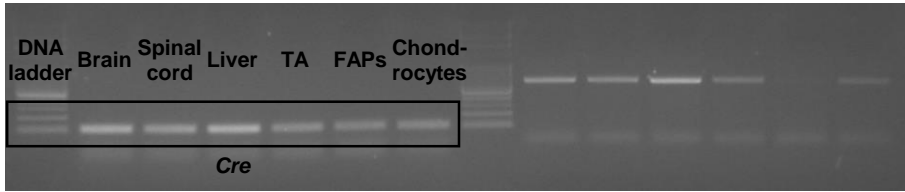

Figure 2G\_Smn1 F7

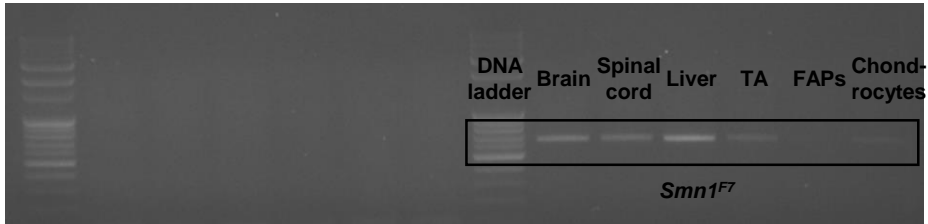

Figure 2G\_Smn1 D7

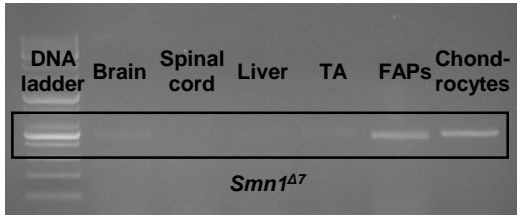

**I**

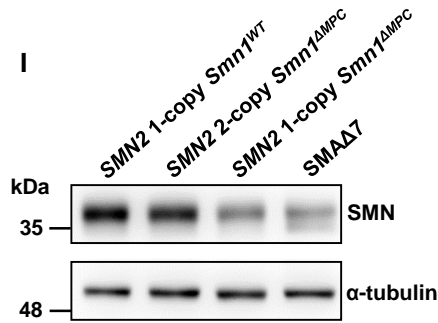

Figure 2I\_anti-alpha-tubulin

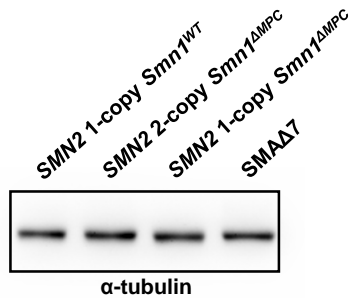

Figure 2I\_anti-SMN

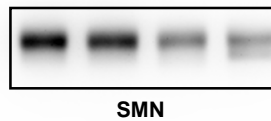

Supplement: Figure 2—source data 2. [file elife-92731-fig2-data2.zip › Figure 2-source data 2.pdf]
